# Plant Biotic and Abiotic Stresses

**DOI:** 10.3390/life14030372

**Published:** 2024-03-12

**Authors:** Hakim Manghwar, Wajid Zaman

**Affiliations:** 1 Lushan Botanical Garden, Jiangxi Province and Chinese Academy of Sciences, Jiujiang 332000, China; 2 Department of Life Sciences, Yeungnam University, Gyeongsan 38541, Republic of Korea

In the complex field of plant science, knowledge of the many difficulties that plants encounter from both living and non-living stresses is essential for maintaining biodiversity and managing natural resources in a sustainable manner, in addition to guaranteeing global food security. Biologic stressors, which are brought on by living things (such as bacteria, viruses, fungus, and insects) and have the potential to cause serious harm, financial losses, and food shortages, are a persistent hazard to plants [1,2,3]. Simultaneously, abiotic stresses impede plant growth, development, and productivity [3,4]. These stresses are caused by non-living elements such as heavy metals, salt, temperature extremes, and water scarcity [5].

The progress made in genetic engineering, molecular biology, and biotechnology has been revolutionary, facilitating the creation of plant types that are resistant to stress [6,7]. These scientific advancements not only improve the ability of crops to withstand challenges but also provide a promising outlook for the implementation of sustainable agriculture methods. The emergence of precision agriculture, driven by Internet of Things (IoT) sensors and artificial intelligence (AI) algorithms, has completely transformed the way plant stressors are monitored and managed [8]. The real-time collection and analysis of data grant the capacity to promptly identify stress indicators, hence permitting timely intervention and implementation of mitigation techniques.

The integrated management strategy is necessary due to the intricate nature of plant stress responses [9]. By integrating conventional agricultural methods with contemporary scientific investigation, it is possible to generate novel and inventive resolutions. For instance, the implementation of crop rotation and the utilization of biopesticides have been shown to effectively mitigate biotic stress [10,11]. Similarly, the incorporation of enhanced irrigation systems and soil amendments has been found to ease abiotic stress [12]. However, the threat of climate change remains significant, intensifying both living and non-living pressures and making it more difficult to combat these difficulties. It is imperative to perform a targeted study to comprehend the intricate relationship between climate change, the interactions between plants and pathogens, and the subsequent reactions to stress.

The need to develop comprehensive models that can accurately forecast the effects of stress and evaluate the efficacy of mitigation techniques cannot be overstated. The effective mitigation of plant biotic and abiotic stresses necessitates a collaborative endeavor involving researchers, farmers, and policymakers. Collaboration is crucial for creating robust agricultural systems that can endure the challenges posed by both living and non-living factors. As we persist in traversing this intricate terrain, the convergence of scientific inquiry and pragmatic implementation will assume a crucial role. By promoting creativity and adopting comprehensive management strategies, the objectives of preserving our crops, guaranteeing food security, and protecting the environment for future generations become more achievable. This research area presents numerous obstacles; however, the potential advantages for both humankind and the environment are extensive and profoundly captivating (Figure 1).

## Figures and Tables

**Figure 1:**
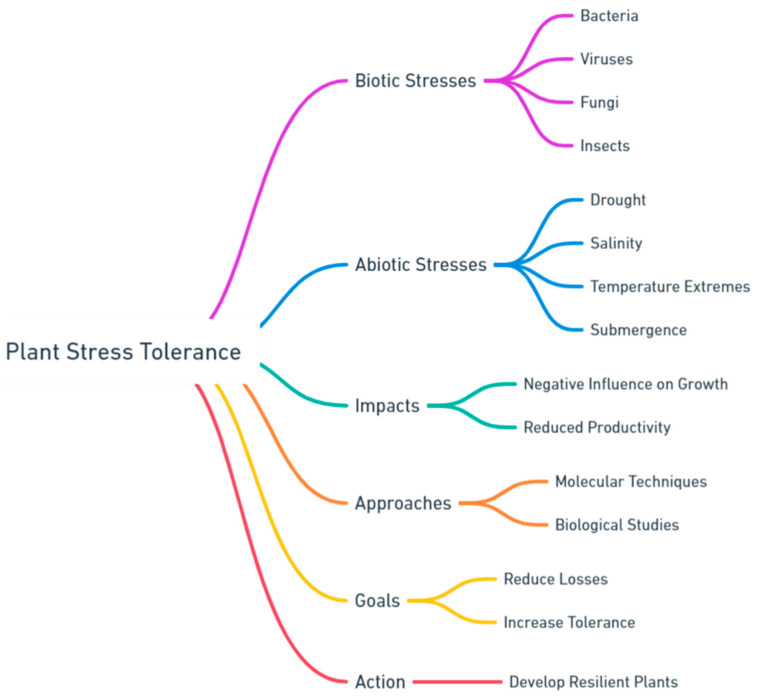
Strategies for enhancing plant resilience to stresses. (Generated by OpenAI’s Whimsical Diagrams for GPT).

## References

[1] Jiménez O.R., Bornemann A.C., Medina Y.E., Romero K., Bravo J.R. (2023). Prospects of biological inputs as a measure for reducing crop losses caused by climate change effects. J. Agric. Food Res..

[2] Balla A., Silini A., Cherif-Silini H., Chenari Bouket A., Moser W.K., Nowakowska J.A., Oszako T., Benia F., Belbahri L. (2021). The threat of pests and pathogens and the potential for biological control in forest ecosystems. Forests.

[3] Suzuki N., Rivero R.M., Shulaev V., Blumwald E., Mittler R. (2014). Abiotic and biotic stress combinations. New Phytol..

[4] Bechtold U., Field B. (2018). Molecular mechanisms controlling plant growth during abiotic stress. J. Exp. Bot..

[5] Rathod A., Verma N.S. (2023). Impact of Abiotic Stress on Agronomical Crops. Frontiers of Agronomy.

[6] Cabello J.V., Lodeyro A.F., Zurbriggen M.D. (2014). Novel perspectives for the engineering of abiotic stress tolerance in plants. Curr. Opin. Biotechnol..

[7] Liu W., Yuan J.S., Stewart C.N. (2013). Advanced genetic tools for plant biotechnology. Nat. Rev. Genet..

[8] Shaikh T.A., Rasool T., Lone F.R. (2022). Towards leveraging the role of machine learning and artificial intelligence in precision agriculture and smart farming. Comput. Electron. Agric..

[9] Zia R., Nawaz M.S., Siddique M.J., Hakim S., Imran A. (2021). Plant survival under drought stress: Implications, adaptive responses, and integrated rhizosphere management strategy for stress mitigation. Microbiol. Res..

[10] Roberts D.P., Mattoo A.K. (2018). Sustainable agriculture—Enhancing environmental benefits, food nutritional quality and building crop resilience to abiotic and biotic stresses. Agriculture.

[11] Mishra R.K., Bohra A., Kamaal N., Kumar K., Gandhi K., Gk S., Saabale P.R., Sj S.N., Sarma B.K., Kumar D. (2018). Utilization of biopesticides as sustainable solutions for management of pests in legume crops: Achievements and prospects. Egypt. J. Biol. Pest Control.

[12] Imran S., Sarker P., Hoque M.N., Paul N.C., Mahamud M.A., Chakrobortty J., Tahjib-Ul-Arif M., Latef A.A.H.A., Hasanuzzaman M., Rhaman M.S. (2022). Biochar actions for the mitigation of plant abiotic stress. Crop Pasture Sci..

